# Time series sightability modeling of animal populations

**DOI:** 10.1371/journal.pone.0190706

**Published:** 2018-01-12

**Authors:** Althea A. ArchMiller, Robert M. Dorazio, Katherine St. Clair, John R. Fieberg

**Affiliations:** 1 Department of Fisheries, Wildlife and Conservation Biology, University of Minnesota, St. Paul, MN, United States of America; 2 U.S. Geological Survey, Wetland and Aquatic Research Center, Gainesville, FL, United States of America; 3 Department of Mathematics and Statistics, Carleton College, Northfield, MN, United States of America; Odum School of Ecology, University of Georgia, UNITED STATES

## Abstract

Logistic regression models—or “sightability models”—fit to detection/non-detection data from marked individuals are often used to adjust for visibility bias in later detection-only surveys, with population abundance estimated using a modified Horvitz-Thompson (mHT) estimator. More recently, a model-based alternative for analyzing combined detection/non-detection and detection-only data was developed. This approach seemed promising, since it resulted in similar estimates as the mHT when applied to data from moose (*Alces alces*) surveys in Minnesota. More importantly, it provided a framework for developing flexible models for analyzing multiyear detection-only survey data in combination with detection/non-detection data. During initial attempts to extend the model-based approach to multiple years of detection-only data, we found that estimates of detection probabilities and population abundance were sensitive to the amount of detection-only data included in the combined (detection/non-detection and detection-only) analysis. Subsequently, we developed a robust hierarchical modeling approach where sightability model parameters are informed only by the detection/non-detection data, and we used this approach to fit a fixed-effects model (FE model) with year-specific parameters and a temporally-smoothed model (TS model) that shares information across years via random effects and a temporal spline. The abundance estimates from the TS model were more precise, with decreased interannual variability relative to the FE model and mHT abundance estimates, illustrating the potential benefits from model-based approaches that allow information to be shared across years.

## Introduction

Long-term monitoring of animal populations is necessary for understanding the impacts of management actions, global change, and animal diseases. Measurement error, resulting from difficulties in observing or detecting individuals, is common when surveying animal populations, and typically requires specialized data collection protocols and associated models to obtain unbiased estimates of ecological parameters [1]. A variety of methods have been developed to account specifically for imperfect detection when estimating abundance from animal surveys. Distance sampling methods, for example, assume the probability of detection can be modeled as a function of the distance between individuals and the observer [2, 3]. By collecting additional data (distances between individuals and observers), one can fit models that correct for imperfect detection in presence-only (or more aptly “detection-only”) data. Alternatively, marking individuals with devices such as radiocollars provides an opportunity to quantify detection probability by creating “capture histories,” records of when animals were and were not detected. Such data are commonly referred to as presence-absence data, although here we will refer to such data by the more appropriate term “detection/non-detection data.”

Steinhorst and Samuel [4] developed a modified Horvitz-Thompson (mHT) estimator of animal abundance that allows information from detection/non-detection data to be applied to subsequent surveys with detection-only data. Specifically, logistic regression models are fit to detections and non-detections of marked individuals and then used to estimate inclusion probabilities for individuals observed during detection-only surveys; these models are typically referred to as “sightability models” [5]. Rather than assume that detection probabilities are consistent through time and space, sightability models assume that the *relationship* between detection and the sightability model covariates is consistent. In other words, sightability models were developed to account for situations where animals might be distributed on the landscape differently from year to year, or even seasonally, by directly relating visibility bias to covariates that are measured at the time of the surveys. The mHT estimator has been applied to many wildlife species in various geographies, including desert bighorn sheep (*Ovis canadensis*) in southwestern Arizona, USA [6], bowhead whales (*Balaena mysticetus*) in the Bering-Chukchi-Beaufort Seas [7], and moose (*Alces alces*) in Minnesota, Wyoming, Washington, Alaska, USA and Alberta, Canada [8–13]. In northeastern Minnesota, the Minnesota Department of Natural Resources (DNR) has used a sightability model, fit to detection/non-detection data collected from radiocollared moose between 2004 and 2007, to adjust for detection probabilities for moose groups during subsequent annual operational surveys (i.e., detection-only data) between 2005 and 2017 (see for example DelGiudice [13]). This approach should correct for seasonal or annual shifts in animal distributions, such as the shift of moose into visibility-reducing conifer cover during winter in Minnesota [14].

Fieberg *et al*. [8] developed a fully model-based abundance estimator, using sightability data, as an alternative to the traditional design-based mHT estimator of Steinhorst and Samuel [4]. Using two years of Minnesota moose survey data, Fieberg *et al*. [8] demonstrated that the model-based approach provided moose population estimates that were similar to the mHT. Unlike the mHT approach, however, which assumes that the counts from each year and plot are independent, the model-based approach allowed for time series modeling of multiyear survey counts and promised to open the door to further spatiotemporal modeling of animal populations. For instance, a model-based approach offers the potential to share information across time or space by including random effects or deterministic spatial or temporal trends in model parameters that influence population abundance.

The main objective of our study was to extend the model-based approach developed by Fieberg *et al*. [8] to allow for time series modeling of animal abundance, using an application to Minnesota moose survey data as a motivating example. Unfortunately, extending the model-based approach to multiple years of operational survey data proved to be more challenging than we originally expected. As will be discussed below, we found that implementing the model-based approach with more than two years of operational survey data caused model trends to change in unrealistic ways, restricting the utility of the Fieberg *et al*. model. We developed and present here a hierarchical model that is robust to this issue, allowing us to fit models to moose aerial survey data collected from 2005 to 2016. We show how models that share information across years, through the combination of random effects and a temporal spline, result in estimates with increased precision and smoother time trends relative to the traditional mHT estimator or a fixed-effects model with year-specific parameters.

In the following sections, we first describe the underlying data structures, the sightability model assumptions, and the mHT estimator. Then, we describe the model-based estimator from Fieberg *et al*. [8] and illustrate how it was expanded to jointly model multiple years of data. We then illustrate the problem we encountered, i.e., “sensitivity” of the sightability model parameters to the amount of detection-only data, and describe the hierarchical modeling solution. With this solution in place, we develop a model that allows information sharing across years and demonstrate its utility.

## Data

The Minnesota DNR conducted four years of sightability trials using radiocollared individuals from 2004 through 2007 and has conducted annual operational (detection-only) surveys since 2005. For both types of surveys, teams of two observers plus the pilot flew along east-west transects spaced at 0.5 km in helicopters (Bell OH-58A, Bell Helicopter Textron, Fort Worth, TX) during mid-winter when snow depth was ≥ 20 cm. We considered sightability trial data (i.e., detection/non-detection data; *n* = 124 independent sightings) from 2005 through 2007 and operational survey data (i.e., detection-only data; *n* = 1922 independent sightings) from 2005 through 2016 for this study. All data and analysis programs are openly available from the Data Repository for the University of Minnesota at https://doi.org/10.13020/D6N30B.

More complete survey information can be found in Giudice *et al*. [11] and Fieberg and Lenarz [15], but briefly, during sightability trials, helicopter teams flew along transects and identified all visible moose within rectangular plots. Whenever they encountered a radiocollared moose, they recorded the number of moose nearby (i.e., group size) and the amount of screening cover within approximately 10 m of the first animal seen (percent of “visual obstruction”). Other covariates were also recorded, but visual obstruction has been identified as the most suitable covariate for modeling detection probabilities for Minnesota moose surveys [11], and it is the only covariate that we consider for this study. During the sightability trials, when radiocollared moose were not detected during the initial transect survey, the helicopter team subsequently located that moose group via VHF telemetry and recorded the same suite of information. Sightability trial plots were 4.0 x 4.3 km in size and were delineated in such a way as to contain at least one radiocollared moose group most of the time. The resultant sightability trial data consisted of 65 non-detection records and 59 detection records.

The protocol for the annual operational surveys was similar to the sightability trial protocol, except the helicopter team recorded all observed moose regardless of whether or not they were radiocollared, and they did not relocate any radiocollared moose after surveying each plot. Thus, the operational surveys resulted in detection-only data. The operational survey plots were 8.04 x 4.29 km and stratified based on expected moose density (Stratum 1: ≤ 7 moose km^-2^; Stratum 2: 8-20 moose km^-2^; Stratum 3: ≥ 21 moose km^-2^) [15]. We ignored a fourth stratum comprised of nine plots, which were designated in 2012 to look specifically at the relationship between moose distribution and habitat management. Surveyed plots were selected each year for the operational survey with a stratified random sampling design, in which approximately 20% of Stratum 3 plots were sampled, approximately 11% of Stratum 2 plots were sampled and approximately 6% of Stratum 1 were sampled. Plot stratification is reviewed every year and updated to reflect land use changes, past survey results, and expert opinion.

## Sightability model and the modified Horvitz-Thompson estimator

For the sightability data, let:

$$\begin{array}{ll} R= & \text{the}\;\text{number}\;\text{of}\;\text{sightability}\;\text{trials}\;\left(\text{here}\;R=124\right)\\ z_{l}= & \text{a}\;\text{random}\;\text{variable}\;\text{equal}\;\text{to}\;1\;\text{when}\;\text{the}\;l\text{th}\;\text{group}\;\text{is}\;\text{detected}\;\text{and}\;0\;\text{otherwise}\\ & \left(l=1,2,\ldots ,R\right)\\ x_{l}= & \text{the}\;\text{percent}\;\text{visual}\;\text{obstruction}\;(\text{from}\;0\;\text{to}\;1)\;\text{associated}\;\text{with}\;\text{the}\;l\text{th}\;\text{group}\\ & \left(l=1,2,\ldots ,R\right)\\ g_{l}= & \text{the}\;\text{probability}\;\text{of}\;\text{detecting}\;\text{the}\;l\text{th}\;\text{group}\;(l=1,2,\ldots ,R) \end{array}$$

For each year *t* of operational survey data (*t* = 2005, 2006, …, 2016), let:

$$\begin{array}{ll} H_{t}= & \text{the}\;\text{number}\;\text{of}\;\text{strata}\;\left(\text{here}\;H_{t}=3\right)\\ N_{h,t}= & \text{the}\;\text{total}\;\text{number}\;\text{of}\;\text{plots}\;\text{in}\;\text{stratum}\;h\\ n_{h,t}= & \text{the}\;\text{number}\;\text{of}\;\text{sampled}\;\text{plots}\;\text{in}\;\text{stratum}\;h\\ M_{h,i,t}= & \text{the}\;\text{number}\;\text{of}\;\text{animal}\;\text{groups}\;\text{in}\;\text{plot}\;i\;\text{located}\;\text{in}\;\text{stratum}\;h\\ & \left(\text{an}\;\text{unknown}\;\text{that}\;\text{has}\;\text{to}\;\text{be}\;\text{estimated}\right)\\ m_{h,i,t}= & \text{the}\;\text{number}\;\text{of}\;\text{detected}\;\text{animal}\;\text{groups}\;\text{in}\;\text{plot}\;i\;\text{located}\;\text{in}\;\text{stratum}\;h\\ y_{h,i,j,t}= & \text{the}\;\text{number}\;\text{of}\;\text{animals}\;\text{in}\;\text{the}\;j\text{th}\;\text{group}\;\text{in}\;\text{plot}\;i\;\text{located}\;\text{in}\;\text{stratum}\;h\\ x_{h,i,j,t}= & \text{the}\;\text{percent}\;\text{visual}\;\text{obstruction}\;(\text{from}\;0\;\text{to}\;1)\;\text{of}\;\text{the}\;j\text{th}\;\text{group}\;\text{in}\;\text{plot}\;i\\ & \text{located}\;\text{in}\;\text{stratum}\;h\\ g_{h,i,j,t}= & \text{the}\;\text{probability}\;\text{of}\;\text{detecting}\;\text{the}\;j\text{th}\;\text{group}\;\text{in}\;\text{plot}\;i\;\text{located}\;\text{in}\;\text{stratum}\;h \end{array}$$

Note, the subscripts *h*, *i*, and *j* refer to data from the operational surveys whereas the subscript *l* refers to data from the sightability trials.

Sightability models assume the probability, *g*_*l*_, of detecting an animal group *l* can be modeled using logistic regression with one or more covariates (*x*_*l*_):

$$\begin{array}{c} \text{logit}\left(g_{l}\right)=\beta_{0g}+\beta_{1g}x_{l} \end{array}$$ (1)

We consider a single covariate (*x*_*l*_) measuring the percent of visual obstruction associated with each animal group (see Giudice *et al*. [11]).

For the mHT estimator, the fitted sightability model is used to adjust for unobserved animals in operational surveys, assuming the model remains appropriate (i.e., the factors influencing detection have not changed). (The mHT estimator also assumes a closed population, independently observed groups of animals, and a statistically valid survey design [4].) First, the logistic regression model (Eq 1) is fit using *x*_*l*_ and *z*_*l*_. Then, the model parameter estimates, ${\hat{\beta }}_{0g}$ and ${\hat{\beta }}_{1g}$, and their covariance matrix are used to estimate a sightability inflation factor. The year-specific mHT estimator of abundance (${\hat{\tau }}_{t}^{\text{mHT}}$) is then given by:

$$\begin{array}{c} {\hat{\tau }}_{t}^{\text{mHT}}=\sum_{h=1}^{H_{t}}\sum_{i=1}^{n_{h,t}}\sum_{j=1}^{m_{h,i,t}}\frac{y_{h,i,j,t}{\hat{\theta }}_{h,i,j,t}}{\pi_{h,i,t}} \end{array}$$

where ${\hat{\theta }}_{h,i,j,t}$ is the sightability inflation factor (${\hat{\theta }}_{h,i,j,t}\approx 1/{\hat{g}}_{h,i,j,t}$) and *π*_*h*,*i*,*t*_ is the probability that the *ith* plot is sampled in year *t*. More details can be found in Fieberg [5], Giudice *et al*. [11], and Steinhorst and Samuel [4].

In this study we used the *Sight.Est* function in the R package *SightabilityModel* [5, 16] to estimate abundance with the mHT estimator. We specified the variance option “Wong,” log-normally distributed confidence intervals, and *α* = 0.10 [5].

## Review of Fieberg *et al*. (2013) model-based approach

Hierarchical models, such as the Fieberg *et al*. [8] model, are often based on two conditional and partially observable random processes [17–19]. Here, the first process represents the abundance of animals within each plot, which is determined by the number and size of animal groups present. The second process represents the detection process, which is conditional on the presence of animals (and the plot being included in the survey).

### Population model

The abundance in year *t* is a function of the number of animal groups (*M*_*h*,*i*,*t*_) and the size of each animal group (*y*_*h*,*i*,*j*,*t*_). The number of animal groups per plot is an unknown parameter, which creates a computational challenge when using Markov chain Monte Carlo (MCMC) methods to fit the model. Specifically, the dimension of *y*_*h*,*i*,*j*,*t*_, and any other variables defined at the group level (e.g., *x*_*h*,*i*,*j*,*t*_), would potentially change with each new iteration of the MCMC sampler. This problem is commonly solved by using parameter-expanded data augmentation [20–22]. Data augmentation offers a general approach to abundance estimation in a Bayesian framework by creating a zero-inflated version of the complete dataset likelihood (taking into account a large number of unobserved data records) in order to fix the dimension of the parameter space. In this case, Fieberg *et al*. [8] augmented each plot with *B*_*h*_ − *m*_*h*,*i*,*t*_ unobserved records, where *B*_*h*_ = (40, 60, 100) for plots in stratum 1, 2, and 3, respectively; the actual values of *B*_*h*_ are not important as long as *B*_*h*,*i*,*t*_ ≫ *M*_*h*,*i*,*t*_. The unobserved records represent animal groups that were not a part of the population or were present but were not detected. Fieberg *et al*. [8] represented this partially observed state (part of the population or not) with a binary indicator variable *q*_*h*,*i*,*j*,*t*_ ∼ Bernoulli(*ψ*_*h*,*i*,*t*_); here, *ψ*_*h*,*i*,*t*_ is the probability that each group in plot *i* in stratum *h* belongs to the “true” population.

Fieberg *et al*. [8] modeled *ψ*_*h*,*i*,*t*_ for all plots in the operational survey data using a Beta($a_{h,t}^{\psi },b_{h,t}^{\psi }$) distribution that varied by year and stratum. Here, we will apply the alternative parameterization from the Fieberg *et al*. [8] supplementary information, which leads to better mixing of the MCMC chains. Specifically, we induced priors for $a_{h,t}^{\psi }$ and $b_{h,t}^{\psi }$ by specifying hyperpriors ($\mu_{h,t}^{\psi }$ and $\rho_{h,t}^{\psi }$), where $\mu_{h,t}^{\psi }$ is the expected value of *ψ*_*h*,*i*,*t*_, $E\left[\psi_{h,i,t}\right]=\mu_{h,t}^{\psi }=a_{h,t}^{\psi }/\left(a_{h,t}^{\psi }+b_{h,t}^{\psi }\right)$, and $\rho_{h,t}^{\psi }=\left(a_{h,t}^{\psi }+b_{h,t}^{\psi }\right)$:

$$\begin{array}{l} \mu_{h,t}^{\psi }∼\text{Uniform}\left(0.01,0.99\right)\\ \rho_{h,t}^{\psi }∼\text{N}\left(5,1\right),\text{truncated}\;\text{to}\;\left(0.01,10\right)\\ a_{h,t}^{\psi }=\mu_{h,t}^{\psi }\rho_{h,t}^{\psi }\\ b_{h,t}^{\psi }=\rho_{h,t}^{\psi }-\mu_{h,t}^{\psi }\rho_{h,t}^{\psi } \end{array}$$

For the model-based estimator, we also need to make a parametric assumption about the distribution of *y*_*h*,*i*,*j*,*t*_. Fieberg *et al*. [8] modeled moose group sizes using a shifted Poisson distribution:

$$\begin{array}{c} y_{h,i,j,t}-1∼\text{Poisson}\left(\lambda_{h,t}\right) \end{array}$$

and specified N(0, 3.16) priors for each log(λ_*h*,*t*_). Note that counts, such as *y*_*h*,*i*,*j*,*t*_, can be modeled with any discrete positive distribution (e.g., Poisson or negative binomial). Also, one could use a truncated distribution to ensure *Pr*(*y*_*h*,*i*,*j*,*t*_ = 0) = 0 rather than a shifted distribution as above.

### Detection model

In their model-based approach, Fieberg *et al*. [8] assumed that the same sightability model applied to both the sightability and operational data sets and thus they jointly modeled the sightability model parameters, *β*_0*g*_ and *β*_1*g*_, with data from both surveys:

$$\begin{array}{cc} \text{logit}(g_{l}) & =\beta_{0g}+\beta_{1g}x_{l},\text{and}\\ \text{logit}(g_{h,i,j,t}) & =\beta_{0g}+\beta_{1g}x_{h,i,j,t} \end{array}$$

The complete data likelihood also requires a model for the marginal distribution of *x*_*h*,*i*,*j*,*t*_ in the operational survey data. Since *x*_*h*,*i*,*j*,*t*_ values are between 0 and 1, Fieberg *et al*. [8] chose a Beta distribution. Here, we will use the same parameterization for $x_{h,i,j,t}∼\text{Beta}\left(a_{h,t}^{x},b_{h,t}^{x}\right)$ as we used for *ψ*_*h*,*i*,*t*_ (i.e., we specify priors for $\mu_{h,t}^{x}$ and $\rho_{h,t}^{x}$). We specified Gaussian priors for the sightability model parameters, *β*_0*g*_ ∼ *β*_1*g*_ ∼ *N*(0, 3.16). This specification allowed both the detection/non-detection data from the sightability trials and the detection-only data from the operational surveys to inform the detection process. (We will later see that this becomes problematic.)

Now, let *z*_*h*,*i*,*j*,*t*_ be random variables equal to 1 if the *jth* group was detected and 0 otherwise. Fieberg *et al*. [8] assumed that *z*_*h*,*i*,*j*,*t*_ are independent Bernoulli trials with probability *g*_*h*,*i*,*j*,*t*_ conditional on both visual obstruction (*x*_*h*,*i*,*j*,*t*_) and presence (*q*_*h*,*i*,*j*,*t*_):

$$\begin{array}{c} z_{h,i,j,t}|x_{h,i,j,t},q_{h,i,j,t}∼\text{Bernoulli}\left(g_{h,i,j,t}q_{h,i,j,t}\right) \end{array}$$

### Abundance estimator

Fieberg *et al*. [8] estimated abundance (${\hat{\tau }}_{t}^{\text{JAGS}}$) using the modeled counts (*y*_*h*,*i*,*j*,*t*_) and the presence indicators (*q*_*h*,*i*,*j*,*t*_):

$$\begin{array}{c} {\hat{\tau }}_{t}^{\text{JAGS}}=\sum_{h=1}^{H_{t}}\sum_{i=1}^{N_{h,t}}\sum_{j=1}^{B_{h,i,t}}y_{h,i,j,t}q_{h,i,j,t} \end{array}$$ (2)

Note, the presence indicators, *q*_*h*,*i*,*j*,*t*_, were set to 1 for all observed moose groups and were imputed for all augmented moose groups. For the unsampled plots, Fieberg *et al*. [8] generated samples from the posterior-predictive distributions of *ψ*_*h*,*i*,*t*_, *q*_*h*,*i*,*j*,*t*_, and *y*_*h*,*i*,*j*,*t*_.

## Expansion of Fieberg *et al*. (2013) approach to multiyear surveys

### Fixed-effect model-based estimator

We begin by exploring a fixed-effect, model-based approach for multiyear surveys where sightability model parameters are shared across years, but all other parameters are year-specific:

$$\begin{array}{ll} z_{l}∼ & \text{Bernoulli}\left(g_{l}\right)\\ y_{h,i,j,t}-1∼ & \text{Poisson}\left(\lambda_{h,t}\right)\\ q_{h,i,j,t}∼ & \text{Bernoulli}\left(\psi_{h,i,t}\right)\\ \psi_{h,i,t}∼ & \text{Beta}\left(a_{h,t}^{\psi },b_{h,t}^{\psi }\right)\\ z_{h,i,j,t}|x_{h,i,j,t}q_{h,i,j,t}∼ & \text{Bernoulli}\left(g_{h,i,j,t}q_{h,i,j,t}\right)\\ \text{logit}\left(g_{l}\right)= & \beta_{0g}+\beta_{1g}x_{l}\\ \text{logit}\left(g_{h,i,j,t}\right)= & \beta_{0g}+\beta_{1g}x_{h,i,j,t}\\ x_{h,i,j,t}∼ & \text{Beta}\left(a_{h,t}^{x},b_{h,t}^{x}\right) \end{array}$$

for every year *t* (*t* = 2005, 2006, …, 2016). We used the same prior distributions as described earlier (i.e., each year was treated separately, with no information sharing across years). We refer to this model as the “FE model” for short.

### Temporal model-based estimator

We created a temporally-smoothed, model-based estimator to jointly model all 12 years of operational survey data (2005 through 2016) with random effects and a temporal spline, thereby allowing information to be shared across years. We refer to this approach as the temporal-spline model-based estimator or “TS model” for short. Specifically, we modeled mean stratum- and year-specific group sizes, via λ_*h*,*t*_, using exchangeable random effects:

$$\begin{array}{ll} \text{log}\left(\lambda_{h,t}\right)= & \mu_{h}+\delta_{h,t}\\ \mu_{h}∼ & \text{N}\left(0,3.16\right)\\ \delta_{h,t}∼ & \text{N}\left(0,\sigma_{h}^{2}\right)\\ \sigma_{h}∼ & \text{Uniform}\left(0,3\right) \end{array}$$

for each year *t*.

For the TS model, we modeled the mean of *ψ*_*h*,*i*,*t*_ using a temporal spline with stratum-specific intercepts:

$$\begin{array}{c} \text{logit}\left(\mu_{h,i,t}^{\psi }\right)=\beta_{0\psi }\varphi_{1}+\beta_{1\psi }\varphi_{2}+\beta_{2\psi }\varphi_{3}+\beta_{3\psi }\gamma_{1}+\beta_{4\psi }\gamma_{2}+\beta_{5\psi }\gamma_{3} \end{array}$$ (3)

where *φ*_*h*_ are dummy variables for stratum 1, 2 and 3, respectively, and *γ* are basis vectors from a natural cubic spline. We chose to include stratum-specific intercepts to capture variation in the expected abundance of moose by strata. We derived the basis vectors using the function *ns*() from the *splines* library with three degrees of freedom and knots placed at the default locations (2010 and 2014) based on quantiles of the years of the operational survey [16]. We chose to use three degrees of freedom to allow for a non-linear response of *ψ*_*h*,*i*,*t*_ across years. We used vague N(0, 5) priors for all of the $\mu_{h,i,t}^{\psi }$ regression parameters (e.g., *β*_0*ψ*_, *β*_1*ψ*_, …, *β*_5*ψ*_).

We used fixed-effects to model the distribution of *x*_*h*,*i*,*j*,*t*_ in each year rather than allow information about the distribution of visual obstruction to be shared across years. This decision is consistent with the general assumption underlying the sightability model approach—i.e., animals shift in their distribution annually, and it is possible to adjust for the resulting changes in detectability using covariates (i.e., *x*_*h*,*i*,*j*,*t*_).

### Sensitivity to the amount of detection-only data

We initially used the *R2jags* package [23] to implement the FE model with the sightability trial data and varying amounts of the operational survey data collected from 2006 through 2016. We sampled from two chains, each with 40,000 MCMC iterations, with the first 20,000 discarded for burn-in. We retained every other sample, leaving us with 20,000 MCMC iterations to summarize posterior distributions. For this and all subsequent implementations of the model-based approaches, we examined the chains to determine mixing and convergence and verified that $\hat{R}<1.1$ for each model parameter [23]. We calculated abundance as the mean of the posterior distribution of ${\hat{\tau }}_{t}^{\text{JAGS}}$ (Eq 2) with 90% credible intervals based on quantiles of the posterior-predictive distributions.

**Fig 1:**
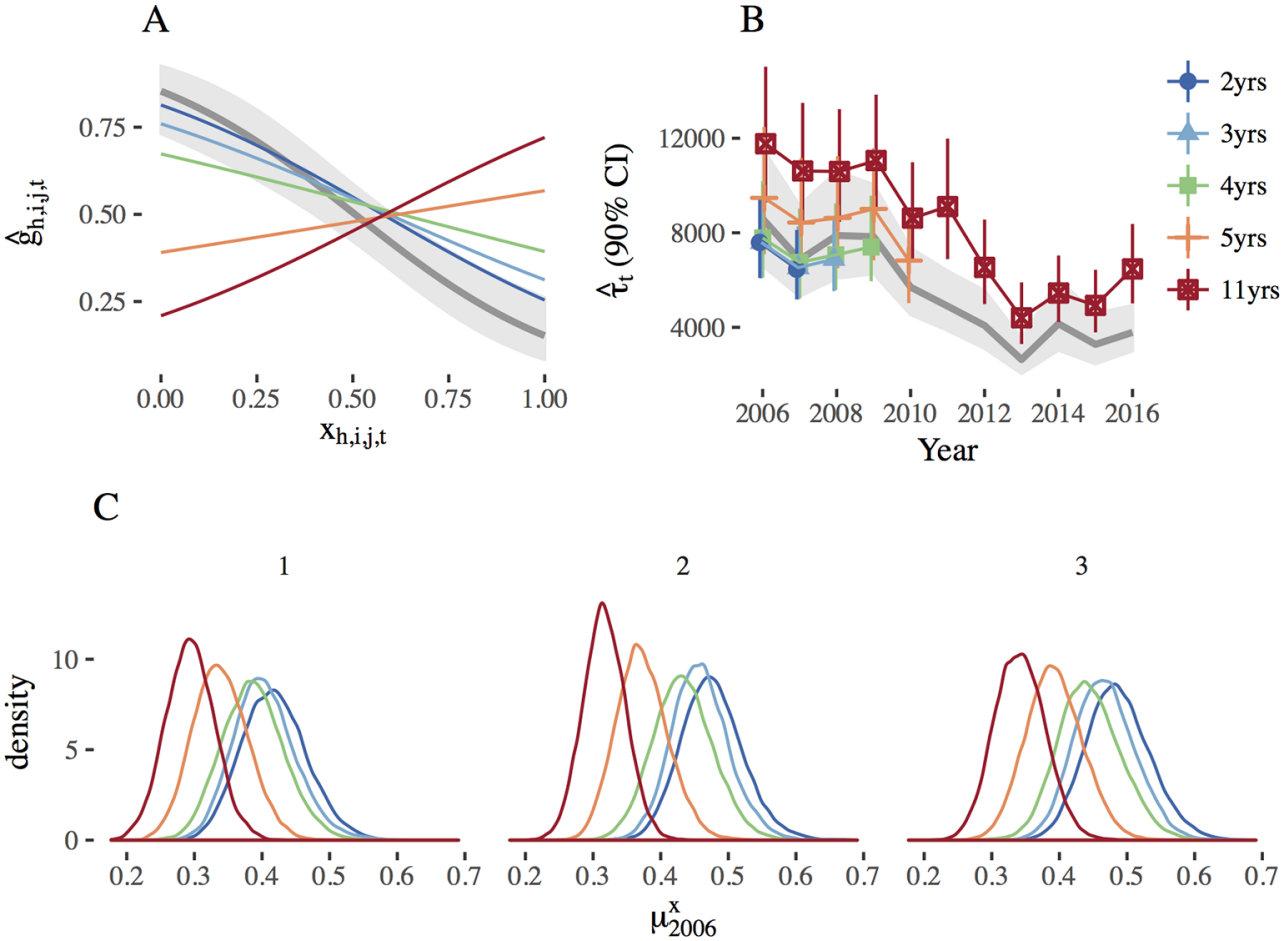
Sensitivity to the amount of detection-only data. A: Sightability model curves demonstrating the relationship between predicted detection probability (${\hat{g}}_{h,i,j,t}$) and visual obstruction (*x*_*h*,*i*,*j*,*t*_) for each moose group *j* in plot *i* in stratum *h* in year *t*, B: abundance estimates ($\hat{\tau }$) by year with 90% credible intervals, and C: the posterior distribution of mean voc by strata *h* in 2006 ($\mu_{2006}^{x}$) for the fixed-effect, joint model-based approach following Fieberg *et al*. [8] with varying numbers of years of operational survey data included in the model. Gray bands and lines in (A) and (B) represent estimates and 90% confidence intervals for the sightability model curve and abundance estimates using the modified Horvitz-Thompson approach, respectively.

When we implemented the FE model in JAGS with multiple years of operational survey data, we discovered that the estimates of the sightability model parameters and abundance estimates were sensitive to the amount of operational survey data included in the analysis (Fig 1). Specifically, the estimated sightability model curve became flattened and eventually switched signs (Fig 1A), and the marginal distributions of *x*_*h*,*i*,*j*,*t*_ shifted towards lower values of visual obstruction with each subsequent year of operational survey data added (e.g., see posteriors of $\mu_{h,2006}^{x}$; Fig 1C). Consequentially, the abundance estimates increased as more years of operational survey data were included (Fig 1B).

When we jointly modeled 2 years of operational survey data (2006 and 2007), the sample sizes for the sightability data, the operational survey data, and the augmented operational survey data were *R* = 124, *m* = 372, and *m*_*aug*_ = 4380, respectively, with a final ratio of sightability data to augmented operational data of 0.028. When we jointly modeled 5 years of operational survey data (2006 to 2010) and the sightability model “flipped”, the ratio of sightability to augmented survey data was 0.011 (*m* = 1039 and *m*_*aug*_ = 11,440). Finally, when 11 years of operational survey data (2006 to 2016) were jointly modeled, the ratio of sightability to augmented operational survey data was 0.005 (*m* = 1922 and *m*_aug_ = 26,960). Considered together with Fig 1, these results seem to suggest that the operational survey data informed the sightability model parameters and the marginal distribution of *x*_*h*,*i*,*j*,*t*_, and eventually the operational survey (detection-only) data overwhelmed the information present in the sightability trial (detection/non-detection) data.

### Model specification

To describe the modeling framework for the model-based approach applied to multiyear surveys, we will use common likelihood notation including brackets “[ ]” to refer to probability density functions and “|” for conditional relationships (e.g., [*a*|*b*, *c*] can be read as the conditional density of *a* given *b* and *c*) [24].

**Fig 2:**
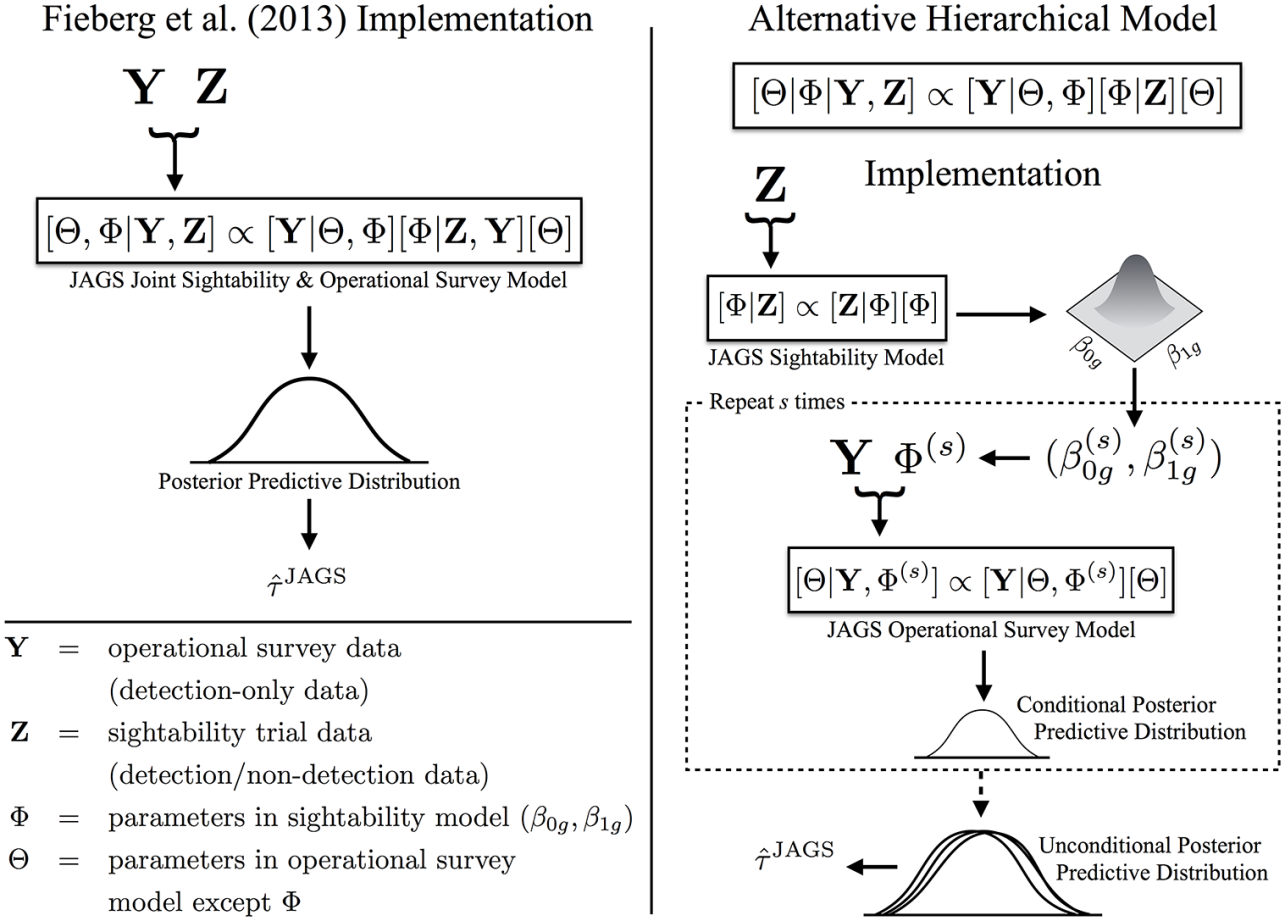
Model comparison diagram. Diagram comparing the original joint hierarchical model of Fieberg *et al*. [8] and the alternative hierarchical model implemented using the two-step approach. The left shows the original, Fieberg *et al*. [8] approach, which jointly models the detection-only data (**Y**) and the detection/non-detection data (**Z**) together. The right shows the alternative, hierarchical model, with parameters estimated by: (1) generating MCMC samples that converge in distribution to [Φ|**Z**] ∝ [**Z**|Φ][Φ]; this is accomplished by fitting the sightability model to **Z** only; (2) sampling from [Φ|**Z**] *s* times to get $\Phi^{\left(s\right)}=\left(\beta_{0g}^{\left(s\right)},\beta_{1g}^{\left(s\right)}\right)$, which we subsequently treat as “data” in step [3]; and (3) generating MCMC samples that converge in distribution to [Θ|**Y**, Φ^(*s*)^] ∝ [**Y**|Θ, Φ^(*s*)^][Θ]. Finally, we pool all posteriors from [3] together to create a pooled, unconditional posterior predictive distribution for estimating abundance (${\hat{\tau }}^{\text{JAGS}}$).

Let Θ represent all of the parameters in the operational survey model except *β*_0*g*_ and *β*_1*g*_, Φ represent the sightability model parameters, *β*_0*g*_ and *β*_1*g*_, **Y** represent the data from the operational surveys (e.g., *m*_*h*,*i*,*j*,*t*_, *y*_*h*,*i*,*j*,*t*_, *x*_*h*,*i*,*j*,*t*_, *z*_*h*,*i*,*j*,*t*_), and **Z** represent the sightability trial data, **Z** = (*z*_1_, *z*_2_, …, *z*_*R*_)′. The unnormalized posterior density of Θ and Φ with our original specification of the FE model is given by:

$$\begin{array}{c} \left[\Theta ,\Phi |Y,Z]\propto [Y|\Theta ,\Phi ][Z|\Phi ][\Phi ][\Theta \right] \end{array}$$ (4)

where [**Y**|Θ, Φ] is the likelihood of the operational survey data, which depends on both Θ and Φ, [**Z**|Φ] is the likelihood for the sightability data, and [Θ] and [Φ] are the prior densities for the parameters. With this specification, the posterior distribution for the sightability model parameters will be informed by both the sightability trial and the operational survey data (Fig 2):

$$\begin{array}{c} \left[\Theta ,\Phi |Y,Z]\propto [Y|\Theta ,\Phi ][\Phi |Z,Y][\Theta \right] \end{array}$$

### Alternative hierarchical model specification

We developed an alternative hierarchical model in which the sightability parameters, *β*_0*g*_ and *β*_1*g*_ are informed by only the sightability data. In this specification, Φ is assumed to be conditionally independent of the operational survey data given the sightability data, i.e.:

$$\begin{array}{c} \left[\Phi |Z,Y]=[\Phi |Z]\propto [Z|\Phi ][\Phi \right] \end{array}$$

In this case, the posterior density of Θ and Φ can be written as:

$$\begin{array}{c} \left[\Theta ,\Phi |Y,Z]\propto [Y|\Theta ,\Phi ][\Phi |Z][\Theta \right] \end{array}$$ (5)

To fit this hierarchical model (Fig 2), we:

1. Generated MCMC samples that converge in distribution to [Φ|**Z**] ∝ [**Z**|Φ][Φ].
2. Randomly sampled *s* values from [Φ|**Z**], giving us Φ^(1)^, Φ^(2)^, …, Φ^(*s*)^.
3. For each Φ^(*s*)^, we used JAGS to generate MCMC samples that converge in distribution to [Θ|**Y**, Φ^(*s*)^] ∝ [**Y**|Θ, Φ^(*s*)^][Θ], by passing Φ^(*s*)^ as “data.”
4. Pooled the samples from [3] across the different values of Φ^(*s*)^ to summarize posteriors with density [Θ, Φ|**Y**, **Z**] (i.e., Eq 5).

To summarize the posterior distributions for step [1] of the two-step approach for implementing the alternative hierarchical model, we ran 2 chains with 10,000 iterations each, a burn-in of 3000 iterations, and a thinning rate of 2, thus providing 7000 MCMC samples to summarize [Φ|**Z**]. To determine an appropriate burn-in period for [Θ|**Y**, Φ^(*s*)^] in step [3], we ran the TS model with two chains with dispersed starting values and examined the resultant trace plots (e.g., S1 Fig). Chains appeared to converge quickly (within approximately 500 iterations) when sightability model parameters were treated as “data” (S1 Fig). Thus for fitting the FE and TS models, we used *s* = 75, and ran 2 chains of 3000 MCMC iterations each with 2000 discarded for burn-in, and a thinning rate of 2 for each sample of Φ^(*s*)^. Pooling the samples from step [3] gave us 75,000 MCMC iterations to summarize the posteriors in step [4].

Our two-step approach to model fitting was motivated by a desire to use existing software, such as JAGS. While JAGS can fit the full hierarchical model (i.e., the Fieberg *et al*. [8] approach) in one step, there is no way that we know of to fit the alternative hierarchical model in one step using JAGS since the model requires the sightability model parameters and operational survey data to be independent given the sightability trial data. The two-step approach, while practical, is less direct and less efficient than if we had derived and used our own MCMC algorithm to fit the model. For example, the unnormalized posterior density function for the sightability model parameters *β*_0*g*_ and *β*_1*g*_ (Φ) depends only on the resightings of moose groups **Z** as follows:

$$\begin{array}{ll} \left[\Phi |Z\right] & \propto \left[\Phi \right][Z|\Phi ]\\ & \propto \left[\Phi \right]\prod_{l=1}^{R}\left[z_{l}|\Phi \right] \end{array}$$

where [*z*_*l*_|Φ] denotes the probability of Bernoulli outcome *z*_*l*_ conditional on detection probability *g*_*l*_ = logit^−1^(*β*_0*g*_ + *β*_1*g*_*x*_*l*_) (see Eq 1). Therefore, if we had constructed a MCMC algorithm, values of *β*_0*g*_ and *β*_1*g*_ would be computed during each iteration of the algorithm simply by sampling a distribution with the above density function. These same values of *β*_0*g*_ and *β*_1*g*_ then would be used to compute the probability of detecting a moose group in the operational survey as follows: *g*_*h*,*i*,*j*,*t*_ = logit^−1^(*β*_0*g*_ + *β*_1*g*_*x*_*h*,*i*,*j*,*t*_). In this way our MCMC algorithm could fit a model of all the data (i.e., from both sightability trials and operational surveys) with joint posterior density equivalent to Eq (5).

## Results

**Fig 3:**
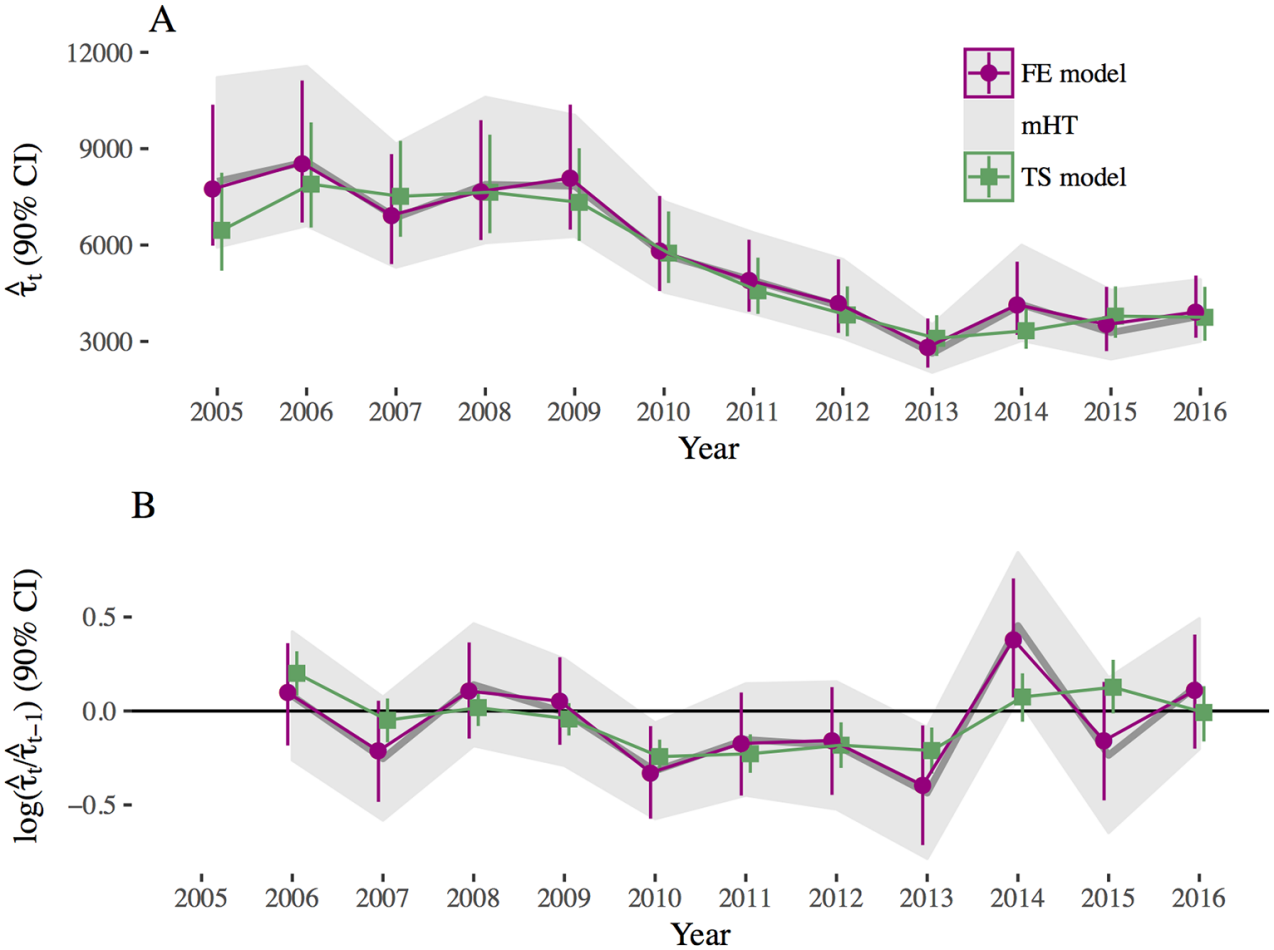
Population estimates by year. A: Population estimates ($\hat{\tau }$) by year *t* and B: the log rate of change of $\hat{\tau }$ between years from the modified Horvitz-Thompson (mHT) approach (grey bands and lines), the fixed-effect, model-based approach (FE model), and the temporal model-based approach (TS model). Error bars are 90% confidence intervals for mHT and 90% credible intervals for the TS and FE models.

Compared with the mHT and fixed-effect model based approaches, the temporal, model-based approach exhibited a smoother, and hence more realistic, trend in population estimates from one year to the next (Fig 3). Estimates from the TS model approach were also more precise (i.e., width of 90% credible intervals were, on average, reduced by 993 animals for TS model compared to mHT) (Fig 3A). Likewise, the log rate of change from one year to the next was also much smoother over time in the TS model compared to the FE model or the mHT estimates (Fig 3B). In particular, the TS model supports a conclusion that the moose population decreased in 2011 and 2012, whereas the mHT and FE model are inconclusive due to larger confidence intervals that overlap zero. Similarly, the mHT and FE model estimators suggest a positive increase in moose abundance in 2014, whereas the TS model supports the notion that moose population numbers have been stable since 2014.

Estimates of λ_*h*,*t*_ were similar when using the FE and TS models, except for the low stratum (i.e., *h* = 1) in 2015 (S2 Fig). The estimate of λ_1,2015_ from the FE model was highly uncertain. By contrast, the exchangeable random effects in the TS model allowed information to be shared across years, resulting in an estimate of λ_1,2015_ with less uncertainty and one that was pulled toward the overall mean. In addition, the use of a natural cubic spline to model changes in the mean *ψ*_*h*,*i*,*t*_ (i.e., $\mu_{h,t}^{\psi }$) resulted in less year-to-year variability and increased the precision of mean *ψ*_*h*,*i*,*t*_ compared to the FE model (S3 Fig).

## Discussion

Studies with marked individuals are expensive and are often cost-prohibitive to apply annually across large spatial scales. Sightability models require an initial investment to develop a model of detection using marked individuals, but are more cost-efficient since the model can then be applied to detection-only surveys in subsequent years, assuming the relationship between covariates and detection probabilities is consistent over time. Hence, the sightability model approach is popular for monitoring many wildlife species, such as moose [8, 9, 11, 15], elk (*Cervus elaphus*) [25, 26], mountain goats (*Oreamnos americanus*) [27, 28], desert bighorn sheep [6], and bowhead whales [7].

The traditional mHT approach, like other survey design-based estimators, can have high sampling variability and can perform poorly when a few large counts occur in a low-density stratum. For instance, the Minnesota DNR conducts annual operational surveys following a stratified random sampling design, assuming that the expected moose density varies between strata, which are defined (and occasionally re-defined) using results from previous surveys, expert opinion, and land cover information [15]. In the Minnesota DNR’s sampling scheme, the high-density stratum (*h* = 3) is sampled at a higher rate than the medium- or low-density strata (*h* = 2 and *h* = 1, respectively), and thus, years with higher mHT uncertainties have been ascribed to atypically high numbers of moose detected in one or more lower-density strata plots (e.g., 2014 mHT abundance) [13]. Similarly, mHT estimates and their standard errors can be sensitive to a small number of observations in heavy cover (animals with low detection probabilities) [29].

Fieberg *et al*. [8] developed the model-based approach with the hope that it might improve precision and also offer additional flexibility when analyzing multi-year surveys. We have shown that a fixed-effects model (with stratum- and year-specific parameters) can produce abundance estimates that closely follow those from the mHT approach. The advantages of the model-based approach are more apparent, however, when models are formulated in a way that allows information to be shared across years. In particular, the TS model provided a smoother, more realistic trend in population through time and abundance estimates with increased precision. To be consistent with the sampling design, the TS model also included stratum-specific intercepts to allow variability in the number of animal groups in each of the strata. Model-based approaches, particularly those with random effects as we illustrate here, can expand the applicability and inference space of design-based estimators (e.g., Royle and Kéry [30]).

With the mHT approach, sightability model parameters are estimated using only the detection/non-detection data. We originally thought that there might be *some* information in the detection-only data that could be used to inform sightability model parameters (e.g., information about the marginal distribution of visual obstruction), and that joint models (e.g., Eq 4) might lead to improved precision for these parameters. We found that estimates of sightability model parameters were sensitive to the amount of detection-only data, but also that estimates became unreliable as additional detection-only data were included in the analysis. Our alternative hierarchical model formulation, fit using a two-step approach, allows sightability model parameters to be informed only by the detection/non-detection data, similar to the mHT approach.

In the joint hierarchical modeling approach of Fieberg *et al*. [8], we generated one or more long Markov Chains, whereas in the two-step approach, we generated several shorter chains, each with a different set of sightability model parameters in step [3]. The two-step modeling approach substantially increased the computational burden required to fit these models (e.g., increased runtime by almost an order of magnitude). There may be ways to make model fitting more efficient. Step [3] requires a burn-in for each new value of Φ^(*s*)^. It might be possible to get better starting values to reduce the burn-in requirement, such as by initially running a longer chain with Φ^(1)^ set to posterior means of [Φ|**Z**], then using posterior means from [Θ|**Y**, Φ^(1)^] as starting values for subsequent model runs. Or, a smaller number of dispersed Φ^(*s*)^ values could be chosen (using quantiles of [Φ|**Z**]), with samples from [Θ|**Y**, Φ^(*s*)^] properly weighted when estimating [Θ, Φ|**Y**, **Z**] in step [4]. The most efficient approach would be to build our own MCMC sampler. However, most ecologists that fit models in a Bayesian framework do so using Bayesian software (e.g., WinBugs, JAGS, or Stan; see preface of Kéry and Royle [1]). The advantages of the two-step approach are that it will likely be easier for most ecologists to adapt to their own applications, and the use of a consistent software platform (e.g., JAGS) may allow for better cross-study comparisons.

In addition to the increased computational load, a potential shortcoming in how we applied our two-step approach is that *s* = 75 may be too small to adequately capture the variability in [Φ|**Z**]. Ideally, we would have used a much larger value of *s*, and saved only the last iteration in step [3]. This would have increased runtime further, but would reduce Monte Carlo Error in our estimates of [Θ|**Y**, **Z**] by more closely mimicking a custom built MCMC algorithm.

It is possible that the sensitivity to the amount of detection-only data is related in some way to prior distributions and/or also the need to specify a distribution for *x*_*h*,*i*,*j*,*t*_ for both observed and unobserved moose groups. Lele *et al*. [31] developed “data cloning” techniques to evaluate model identifiability and to implement frequentist estimators using Bayesian software. These approaches work by making many copies of the data (to swamp out any information coming from the prior distributions), with post-hoc adjustments to account for the effect of data cloning on variance estimators. Initially, we explored the possibility of fitting the original hierarchical model, as in Fieberg *et al*. [8], using JAGS with a “partial data cloning” approach in which we made multiple copies of (only) the detection/non-detection data. This approach also led to robust abundance estimators, as sightability model parameters were then effectively informed using only the detection/non-detection data. However, it was not readily apparent how to adjust variances post-hoc to account for the inflated sample size.

In summary, we have developed a robust model-based approach that allows us to increase the precision and reduce the interannual variability of population abundance estimates from multiyear detection-only surveys when combined with a set of detection/non-detection data. In future work, we hope to develop spatially-explicit models for animal abundance, by incorporating plot-specific landscape characteristics such as habitat diversity, snow depth, or forage availability that might affect animal distribution and abundance (e.g., Michaud *et al*. [32]).

## Supporting information

S1 FigExample JAGS traceplot.Example traceplot for the Eq (3) (e.g., *β*_0*ψ*_, *β*_1*ψ*_, …, *β*_5*ψ*_) parameters in the temporal model-based approach (TS model) run with 2 chains, 4000 MCMC samples, no thinning, and no burn-in. For this plot, MCMC samples were generated with sightability model parameters fixed at ${\hat{\beta }}_{0g}=0.33$ and ${\hat{\beta }}_{1g}=-0.99$.(TIFF)

S2 Fig Distribution of λ_*h*,*t*_ by year and stratum. Median and 90% quantiles of the posterior distributions of λ_*h*,*t*_ for each stratum *h* (top to bottom panels) and year *t* from the fixed-effect, hierarchical model-based estimator (FE model) and the temporal hierarchial model-based estimator (TS model). In the temporal model-based estimator approach, λ_*h*,*t*_ were modeled with exchangeable random effects. Plots were stratified based on expected moose density (Stratum 1: ≤ 7 moose km^-2^; Stratum 2: 8-20 moose km^-2^; Stratum 3: ≥ 21 moose km^-2^).
(TIFF)

S3 FigDistribution of mean *ψ*_*h*,*t*_ by year and stratum.Median and 90% quantiles of the posterior distributions of mean *ψ*_*h*,*t*_ (i.e., $\mu_{h,t}^{\psi }$) for each stratum *h* (top to bottom panels) and year *t* from the fixed-effect, hierarchical model-based estimator (FE model) and the temporal hierarchial model-based estimator (TS model). In the temporal model-based estimator approach, *ψ*_*h*,*t*_ were modeled with a natural cubic regression spline with stratum-specific intercepts. Plots were stratified based on expected moose density (Stratum 1: ≤ 7 moose km^-2^; Stratum 2: 8-20 moose km^-2^; Stratum 3: ≥ 21 moose km^-2^).(TIFF)
