# Supplementary figures and images for: Time series sightability modeling of animal populations
Source: PLoS One. 2018 Jan 12;13(1):e0190706. doi: 10.1371/journal.pone.0190706 (PMC5766105; doi:10.1371/journal.pone.0190706)

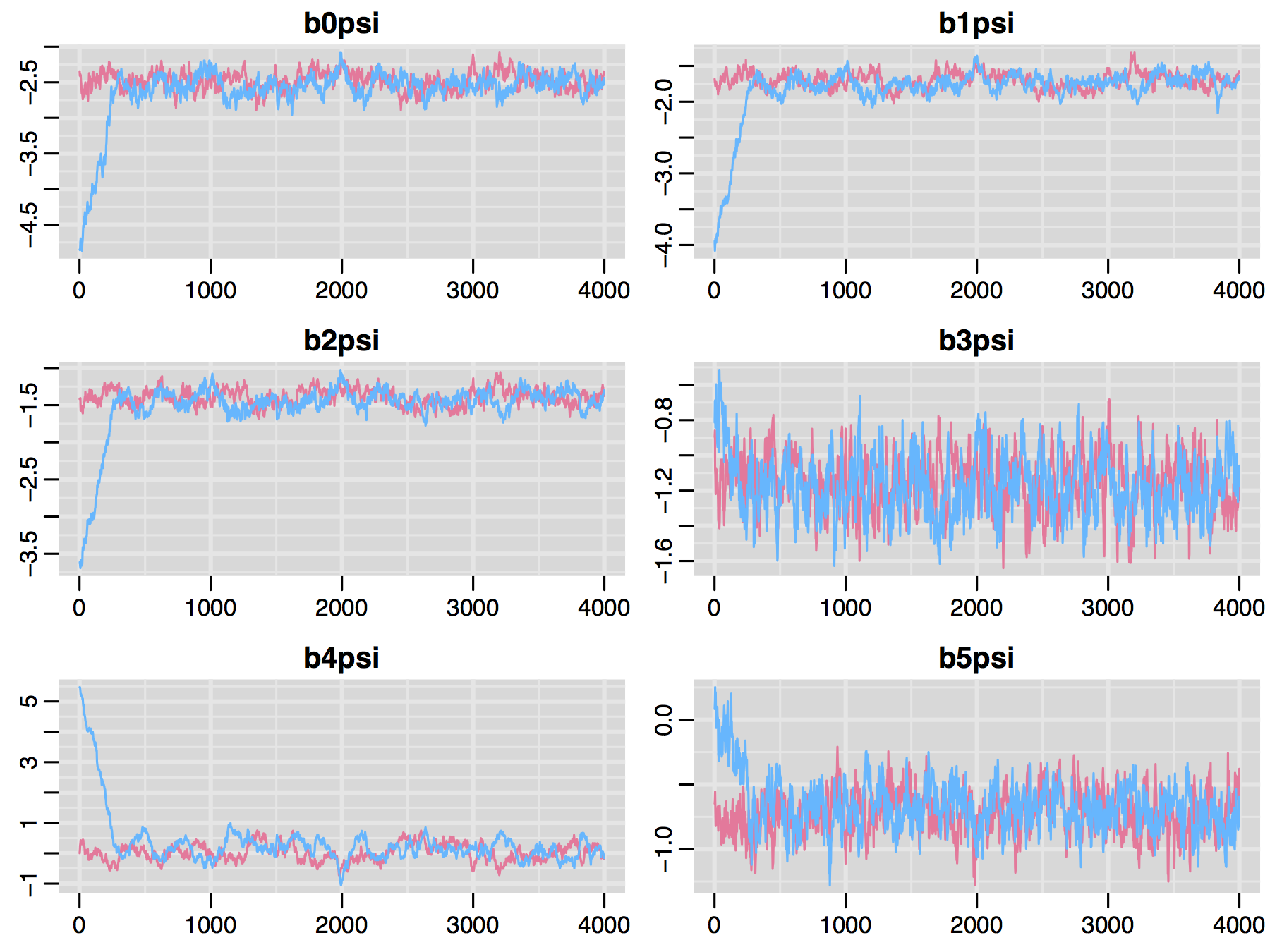

Supplement: S1 Fig — Example traceplot for the Eq (3) (e.g., β0ψ, β1ψ, …, β5ψ) parameters in the temporal model-based approach (TS model) run with 2 chains, 4000 MCMC samples, no thinning, and no burn-in. For this plot, MCMC samples were generated with sightability model parameters fixed at β^0g=0.33 and β^1g=−0.99. (TIFF) [file pone.0190706.s001.tiff]

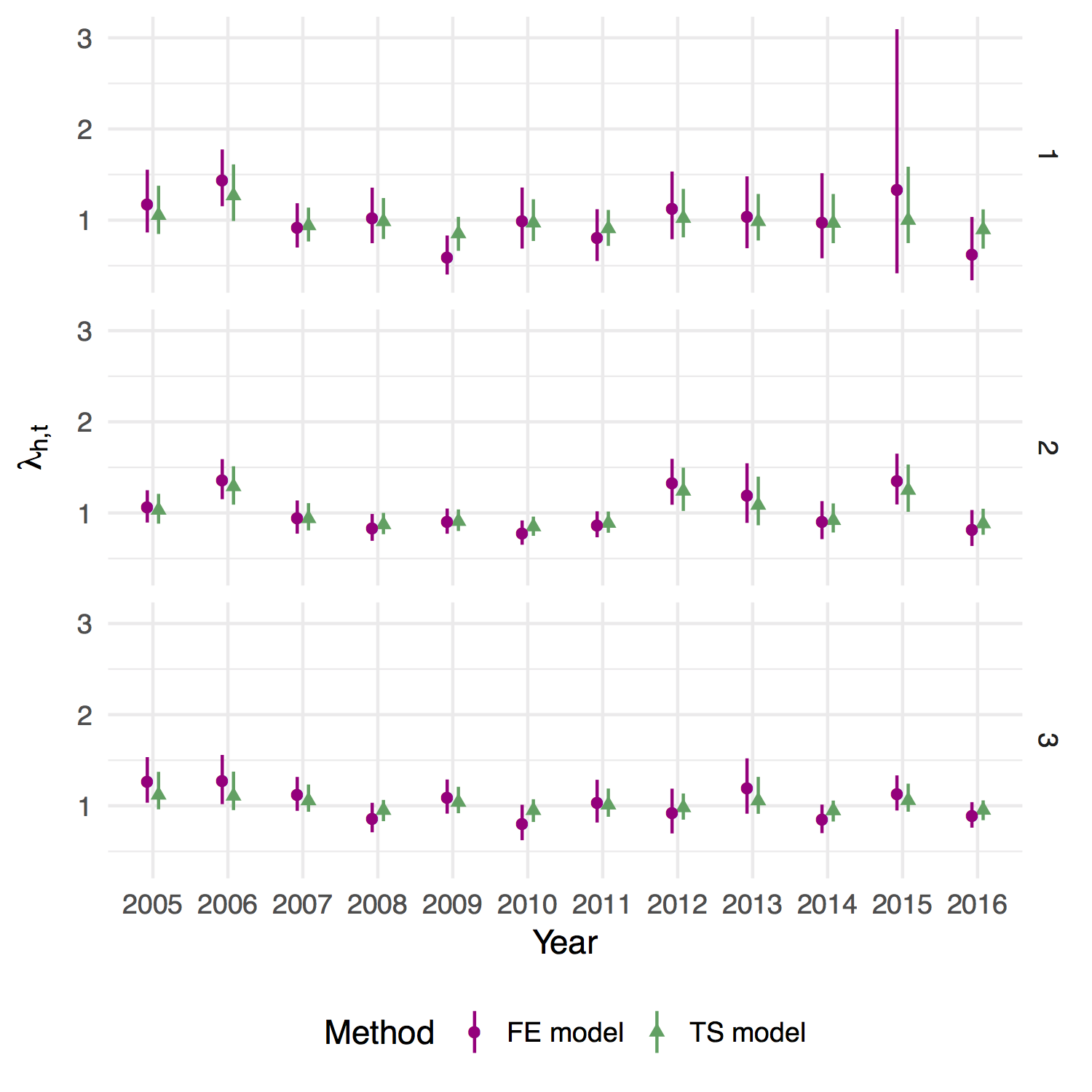

Supplement: S2 Fig — Median and 90% quantiles of the posterior distributions of λh,t for each stratum h (top to bottom panels) and year t from the fixed-effect, hierarchical model-based estimator (FE model) and the temporal hierarchial model-based estimator (TS model). In the temporal model-based estimator approach, λh,t were modeled with exchangeable random effects. Plots were stratified based on expected moose density (Stratum 1: ≤ 7 moose km-2; Stratum 2: 8-20 moose km-2; Stratum 3: ≥ 21 moose km-2). (TIFF) [file pone.0190706.s002.tiff]

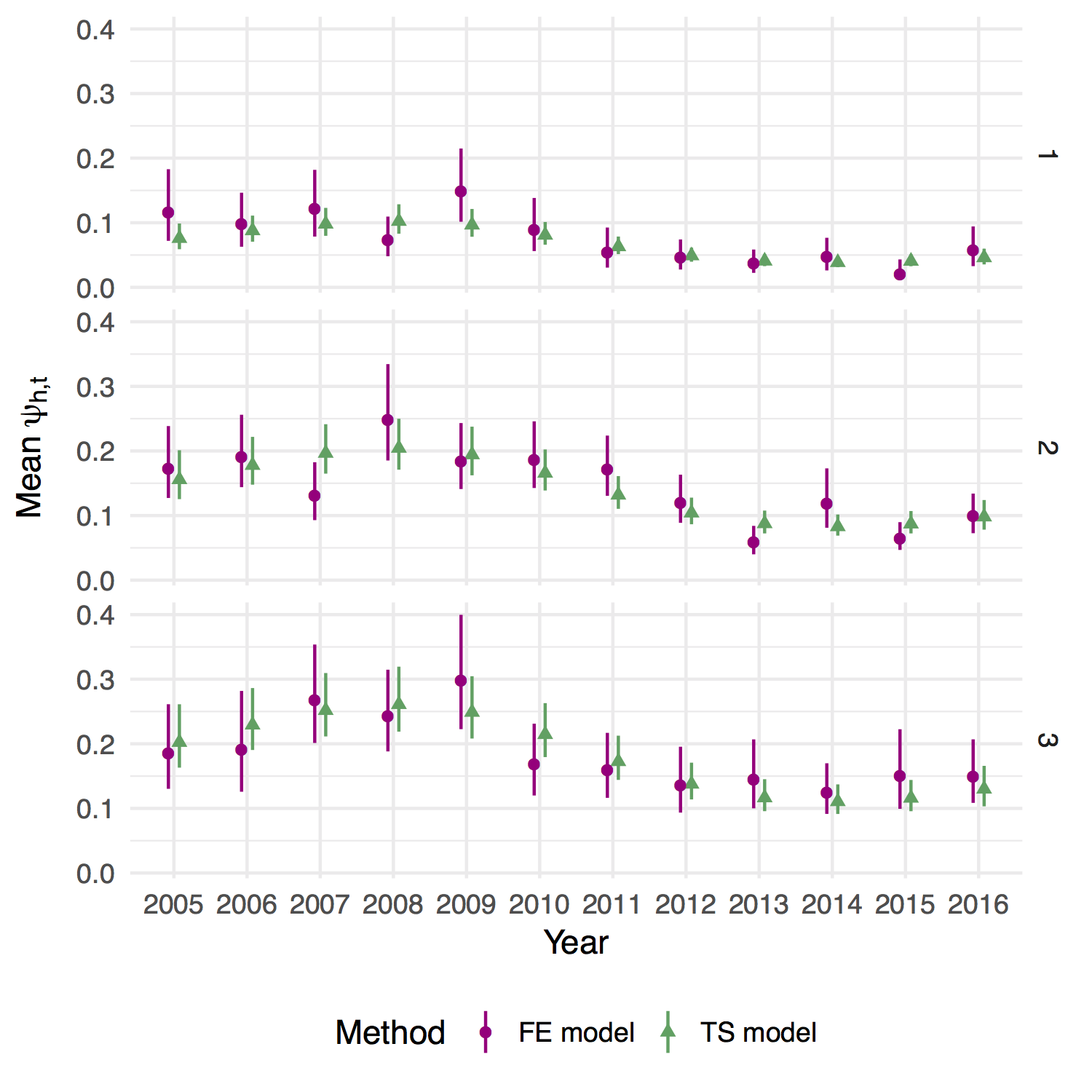

Supplement: S3 Fig — Median and 90% quantiles of the posterior distributions of mean ψh,t (i.e., μh,tψ) for each stratum h (top to bottom panels) and year t from the fixed-effect, hierarchical model-based estimator (FE model) and the temporal hierarchial model-based estimator (TS model). In the temporal model-based estimator approach, ψh,t were modeled with a natural cubic regression spline with stratum-specific intercepts. Plots were stratified based on expected moose density (Stratum 1: ≤ 7 moose km-2; Stratum 2: 8-20 moose km-2; Stratum 3: ≥ 21 moose km-2). (TIFF) [file pone.0190706.s003.tiff]
